# Evaluation of Seed Germination of Six Rare *Stipa* Species following Low Temperature Stress (Cryopreservation)

**DOI:** 10.3390/life13122296

**Published:** 2023-12-02

**Authors:** Ekaterina Sergeevna Osipova, Dmitry Viktorovich Tereshonok, Evgeny Aleksandrovich Gladkov, Sergey Victorovich Evsyukov, Anna Yurievna Stepanova

**Affiliations:** K.A.Timiryazev Institute of Plant Physiology, Russian Academy of Sciences (IPP RAS), 127276 Moscow, Russia; diman_ter_vi@mail.ru (D.V.T.); gladkovu@mail.ru (E.A.G.); evsyukov_2013@mail.ru (S.V.E.); step_ann@mail.ru (A.Y.S.)

**Keywords:** cryopreservation, cryostress, stratification, germination, dormancy, *Stipa* L.

## Abstract

Cryopreservation is one way to preserve rare, endangered species. However, during the cryopreservation process, plant cells undergo considerable stress, which may lead to cell death. In our work, orthodox *Stipa* seeds of six rare species were cryopreserved: *S. sareptana*, *S. ucrainica*, *S. tirsa*, *S. dasyphylla*, *S. adoxa*, and *S. pulcherríma*. Short-term cryopreservation (14 days) stimulated germination of all *Stipa* species studied. Prolonged cryopreservation (70 days and more) decreased the germination of all *Stipa* seeds except *S. sareptana*. The decrease in germination progressed over time as a result of the cumulative stress of cryopreservation rather than the initial stress. To stimulate germination, seeds were stratified and treated with GA_3_, KNO_3_, NaOH, and H_2_O_2_. After four years of seed cryopreservation, it was possible to obtain seedlings of all the *Stipa* species studied with 30 days of stratification and 180 days of germination. After five years of cryopreservation and seed treatment with 30% NaOH for one hour, the best germination was obtained in *S. adoxa* and *S. pulcherrima*. After treatment with 5% H_2_O_2_ for 20 min, the best germination was obtained in *S. sareptana*, *S. ucrainica*, and *S. dasyphylla*. *S. sareptana* seeds germinated in all the aforementioned experiments. *S. sareptana* has a non-deep physiological dormancy and is the most widespread and drought-tolerant *Stipa* species studied. The best habitat adaptation and stress tolerance correlated with this species’cryotolerance. *S. sareptana* was recommended for further cryopreservation, while storage protocols for the other *Stipa* species studied need further improvements.

## 1. Introduction

One of the ways to preserve rare, endangered plant species and unique genotypes is cryopreservation. Cryopreservation is a process of low-temperature storage (−135 °C) of biological objects with the potential of restoring their biological functions after thawing. As a rule, cryopreservation is carried out at −196 °C in a cryobank with liquid nitrogen [1]. The establishment of cryobanks of germplasm: seeds, pollen, embryos, tissue, and cell cultures and other genetic material is included in national programs for the conservation of genetic phytoresources in many countries [2,3,4]. At present, about 400 botanical gardens in the world have seed banks containing more than 300 thousand stored samples [5].

Cryopreservation of hydrated plant cells, tissues, and organs is a very extreme process due to the formation and growth of ice crystals during freezing and dehydration and recrystallisation during subsequent thawing, resulting in cold, mechanical, water, osmotic, and oxidative stress [6]. The formation of ice crystals inside cells can be prevented in several ways: reducing the cooling rate, desiccation, and vitrification. To date, the most widespread dehydration technique is vitrification, which is the conversion of intracellular water into a glassy matrix during ultra-fast freezing. Before vitrification, the explants should be incubated in special solutions such as the Plant Vitrification Solution (PVS) series developed by A. Sakai [7,8]. PVS2 and PVS3 are the most commonly used options [9,10]. Vitrification protocols have been developed and improved over time. Currently, they include encapsulation dehydration [11], encapsulation vitrification [12,13], drip vitrification [14,15], cryoplast protocols [16,17], vacuum infiltration vitrification (VIV) [18,19], and a new cryo-mesh protocol [20]. All cryopreservation protocols are based on dehydration to 10–20% of original weight [21]. However, dehydration may lead to dysfunction in membranes and changes in their barrier properties [22,23,24], as well as destruction of cell organelles [25,26]. As a result of dehydration, the concentration of dissolved compounds, mainly inorganic ions and metabolites with toxic effects, is significantly increased in the cell. Dehydration results in conformational changes in protein macromolecules, causing cross-links within them and, thus, leads to non-reversible biochemical changes [27,28,29]. Changes in protein and lipid properties lead to metabolic disorders, in particular, to suppression of the biochemical and photochemical activity of chloroplasts and mitochondria [30,31], formation of reactive oxygen species (ROS) [32,33,34] and, as aresult, to programmed cell death [35,36]. Thus, the aim of hydrated plant cells’cryopreservationis to minimize the stress effects of dehydration. In some cases, preliminary plant cold acclimation may be helpful [37].

In the case of plant material containing a minimal amount of water (pollen, orthodox seeds), cryopreservation is not that difficult. Such material, previously dried, can be placed directly in liquid nitrogen and then thawed in air under normal conditions [38,39]. Since ultra-low temperatures presumably stop cell metabolism and chemical and physical reactions, it has been suggested that seeds can be stored for decades under such conditions [40]. Despite these assumptions, NCGRP monitoring data show that a decrease in seed viability during cryogenic storage can be detected within 10–25 years for 15 out of 42 seed species stored in liquid nitrogen (−196 °C) [41]. The degradation process progressed over time, demonstrating that the decrease in viability was due to aging stress rather than initial stresses. The authors concluded that despite the glassy matrix forming, slow movement of molecules within the seeds continued. It appears that molecular mobility is an important factor controlling the kinetics of the aging process. The nature and extent of molecular motion vary considerably depending on moisture levels, temperature, and on the hydration levels of the seeds as well [41]. The combination of extreme drying and extreme cooling may result in abnormal temperature responses in aging kinetics [42]. Some authors have reported physiologically dormant seeds having difficulties germinating after cryopreservation [43]. Therefore, the primary objective during cryopreservation of orthodox seeds is to monitor germination consistently. Seed germination after cryopreservation may be influenced by the moisture content levels of the seed, the degree of seed dormancy, the weather conditions during seed formation, the location of collection, and the storage conditions until cryopreservation [41,43].

In our work, orthodox *Stipa* seeds of the following six rare species were cryopreserved: *S. sareptana*, *S. ucrainica*, *S. tirsa*, *S. dasyphylla*, *S. adoxa*, and *S. pulcherríma*. The *Stipa* genera includes more than 300 species of grasses. These perennial grasses have adapted to arid conditions with a low humidity and extreme temperature fluctuations. The caryopsis of *Stipa* is a single structure. It is traditionally referred to as a ‘seed’ in scientific publications [44,45]. The *Stipa* is becoming a rare and protected species due to the development of steppes for agriculture and grazing [46]. At present, *Stipa*, as individual dominants, can be used as indicators of steppe communities and for their classification [47]. In addition, populations of some *Stipa* species may be used as indicators of global climate change [48]. Unfortunately, the system of protected areas cannot ensure the conservation of the biological diversity of *Stipa* species in Russia [49]. In this regard, the aims of our research were to create a seed bank of six *Stipa* species; to test the germination of frozen seeds at time intervals; and to investigate the effect of stratification and different seed stimulants on their germination.

## 2. Materials and Methods

### 2.1. Description of the Stipa Species Studied

**Figure 1:**
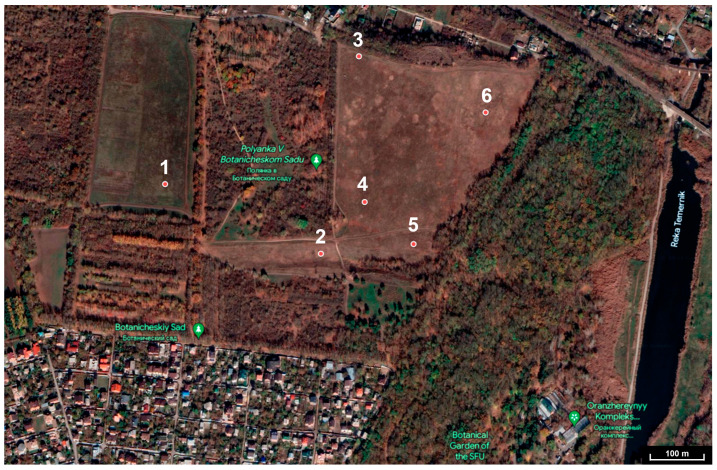
Seed collection site of the *Stipa* samples.

Seeds of six species of *Stipa* were collected in 2015 from the virgin steppes on the territory of the “Botanical Garden of the Southern Federal University” (Rostov-na-Donu) [50]. The Botanical Garden preserves the collection of rare and endangered plant species of the Rostov region and the Don floodplain (Figure 1; Table 1) [51,52]. For our experiments we used three species of *Stipa*: *S. ucrainica* P. Smirn., *S. dasyphylla* (Lindem.) Trautv., and *S. pulcherrima* K. Koch, which are listed in the Red Data Book of the Russian Federation. The other three species: *S. sareptana* A. Becker, *S. tirsa* Steven, and *S. adoxa* Klokov and Ossyczn., are protected by the Red Data Books of various regions of the Russian Federation and other European countries.

### 2.2. Seed Cryopreservation

The seeds were gathered by hand. The long pinnate awns were removed. The palea and lemma were not separated from the caryopsis to avoid damaging the seeds. The collected seeds were placed in labeled paper bags and stored in a dark at room temperature (20–25°C) and 40–60% humidity for several months until the start of the experiment. About 50 seeds of each *Stipa* species were used in the studies.

Biological storage (XB-0.5; manufactured by Ural Compressor Plant, Ekaterinburg, Russian Federation) was used for seed cryopreservation. The seeds were placed in cryoampoules (Nunc, Thermo Fisher Scientific, Waltham, MA, USA) before freezingand then the cryoampoules were placed in liquid nitrogen (N_2_) at −196 °C. The seeds were defrosted at room temperature before the experiment. One cryoampoule of each *Stipa* species was used without refreezing for each experiment.

### 2.3. Seed Viability Testing

The seeds were carefully examined for each experiment and damaged or suspicious seeds were discarded. Seed viability was determined by the TTC (2,3,5-triphenyltetrazolium chloride) method. If seeds are viable, they are actively respiring and reducing triphenyltetrazolium chloride to red formazan. The seeds were placed in Petri dishes on moist filter paper and incubated for 48 hin the dark, at 20–25 °C. The seeds were then immersed in 0.5% TTX solution in phosphate buffer (pH = 7.5) and placed in the dark at 28 °C for 24 h. Seeds were considered viable when their tissues were stained red [53]. Seed viability was determined as the ratio of stained seeds to the total number of seeds and expressed in percents. Seed viability before and after cryopreservation were determined using the TTC method.

### 2.4. Seed Germination Testing

*Stipa* seeds were stored in the dark at room temperature (20–25 °C) for three months from the time of collection until germination. For the germination control test, seeds without pre-sowing treatment were germinated in Petri dishes on moist filter paper for 14 days. Seed germination was determined as the ratio of germinated seeds to the total number of seeds and was expressed in %.

### 2.5. Indication of Dormancy Status

Indication of dormancy status was calculated using Offord’s et al. (2004) equation:

DI (DormancyIndex) = 1—(GI/VI), where
GI (Germination Index)—% of germinated seeds;
VI (Viability Index)—% of viable stained seeds (TTC test)

A higher DI value suggests a higher probability of seed dormancy at that time. If the DI is equal to or greater than 0.4, it indicates dormancy in the seed [54].

### 2.6. Seed Germination after Cryopreservation

#### 2.6.1. Experiment I (the Short-Term Effect of Cryopreservation)

The seeds were divided into two groups: control and test. The control group wasstored in the dark at room temperature for six months from the start of the experiment and then stratified for 42 days. The test group was initially stored in the same way as the control group, but then cryopreserved for 14 days and then stratified for 42 days. Seeds from the control group, which had not been used before, were stored in the dark at room temperature. The remaining seeds were stored in liquid nitrogen. They were thawed as the experiments proceeded.

#### 2.6.2. Experiment II (the Effect of Stratification)

The seeds were separated into four groups. The first group was stored in the dark at room temperature for eight months from the start of the experiment. The second group was also stored in the same way as the first group, but for seven months, followed by 21 days of stratification. The third group was stored in liquid nitrogen for 91 days. The fourth group was also stored in liquid nitrogen for 70 days and then stratified for 21 days.

#### 2.6.3. Experiment III (the Long-Term Effect of Cryopreservation and NaOH Treatment)

The seeds were thawed after 415 days (about 14 months) of storage in liquid nitrogen. The *Stipa* were germinated on Petri dishes for 30 days. Ungerminated seeds were tested for viability using the TTC test. Ungerminated stained seeds were treated with NaOH.

#### 2.6.4. Experiment IV (the Germination Duration)

The seeds were thawed after 1489 days (about 4 years and 1 month) of storage in liquid nitrogen. The seeds were tested for viability using the TTC test. Stratification continued for 30 days. Seed germination was carried out for 12 months.

#### 2.6.5. Experiment V (the Effect of Different Stimulators)

The seeds were thawed after 1951 days (5 years and 4 months) of storage in liquid nitrogen. The seeds were separated into four groups. The seeds of the first group of were treated with H_2_O_2_, while the seeds of the second group were treated with NaOH. The seeds of the third group of were germinated with KNO_3_. The seeds of the fourth groupwere germinated with GA_3_. Unfortunately, the *S. tirsa* seeds were used up at that time and were not included for this experiment.

### 2.7. Seed Stratification

Seed stratification was carried out doors for 3–6 weeks. Seeds were planted in a soil/turf mixture in pots to a 1 cm depth. The pots were then covered with snow for the total stratification period. The air temperature was below 0 °C all the time (from −2 to −15 °C). Thus, we decided to imitate the influence of natural winter conditions on seeds, which last from 4 to 6 months in the Rostov region. At the end of stratification, the seeds were transferred to a greenhouse where they germinated at 40–60% humidity, at 20–25 °C, and 16 h illumination of 2–4 klk per day within 30 days.

### 2.8. Seed Germination Stimulators

To stimulate germination, dry seeds were immersed in 30% sodium hydroxide (NaOH) solution for 1 h or in 5% hydrogen peroxide (H_2_O_2_) solution for 20 min. The seeds were then washed with sterile water and placed in Petri dishes on filter paper moistened with distilled water. In addition, 0.1% potassium nitrate (KNO_3_) and 0.05% gibberellic acid (GA_3_) (Green AgroLab, Orenburg, Russian Federation) were used to stimulate germination. GA_3_ was first dissolved in 2 mL of ethanol and then added to water to the final concentration. As a control, seeds were germinated in Petri dishes on filter paper moistened with distilled water. Petri dishes with seeds were placed under conditions of stable temperature regime (20 ± 2 °C) and illumination (2 klk) at 16 h day (climatic chamber of the phytotron of IPP RAS with automatic air conditioning): Gree, PRC, and fluorescent lighting: LB-40 “OSRAM”, RF. The seeds germinated for 30 days.

### 2.9. Statistics

Statistical analysis was performed using a one-way ANOVA test. The experimental data demonstrated a significant difference at *p* < 0.05.

## 3. Results

### 3.1. Seed Viability Testing

Most of the *Stipa* seeds tested stained red in the TTC test, i.e., they were viable. After cryopreservation, no significant changes in the viability of the seeds were observed, as all the seeds were stained red after thawing.

### 3.2. Seed Germination Testing

For 14 days, only *S. sareptana* seeds (60%) germinated. Other *Stipa* species seeds germinated after one month.

### 3.3. Indication of Dormancy Status

The DI for *S. sareptana* seeds in this case was 0.4 minimal among *Stipa* studied. For the other *Stipa* species, the DI was equal to 1. Therefore, all the *Stipa* seeds were in organic dormancy. Mechanical removal of palea and lemma from caryopsis stimulated the germination of all viable embryos in our experiments. However, this operation usually resulted in catastrophic damage to the caryopsis and unfortunately did not allow for the formation of viable seedlings. Germination after the removal of seed-coats suggests physiological dormancy in *Stipa* seeds.

### 3.4. Seed Germination after Cryopreservation

#### 3.4.1. Experiment I

The first series of experiments evaluated the short-term effect of cryopreservation on seed germination. The experiments showed that the average germination level of the seeds was 40%. The DI was greater than 0.4, indicating that the seeds were in organic dormancy. The average germination increased by 19% (from 40 to 59%) after cryopreservation. The DI after cryopreservation decreased in all *Stipa* species (0.41). In *S. sareptana* and *S. pulcherrima* DI levels were less than 0.4, i.e., the seeds of these species were out of the dormancy (Table 2). The first experiment indicated that short-term cryopreservation increased germination and decreased DI.

#### 3.4.2. Experiment II

The second series of experiments evaluated the effect of stratification on seed germination. The effect of cryopreservation was also tested. Stratification increased the germination levels of *S. sareptana*, *S. dasyphylla*, and *S. pulcherrima*. However, the germination of *S. adoxa* was independent of stratification. The germination of *S. ucrainica* and *S. tirsa* seeds stored at room temperature did not depend on stratification, but the germination of seeds stored in liquid nitrogen increased after stratification. However, on average, stratification increased germination rates (from 14 to 23% and from 24 to 36%). More prolonged stratification is probably needed to stimulate germination in some *Stipa* species (Table 3). The second series of experiments also demonstrated that cryopreservation increased the germination rates of all *Stipa* by 13% (from 23 to 36%). *S. adoxa* did not germinate after 7 and 8 months of storage at room temperature, and this species was only able to germinate after cryopreservation. However, compared to the initial average levels (Table 2), germination after 70 days of cryopreservation decreased in all *Stipa* species except for *S. sareptana* (Table 3).

#### 3.4.3. Experiment III

**Figure 2:**
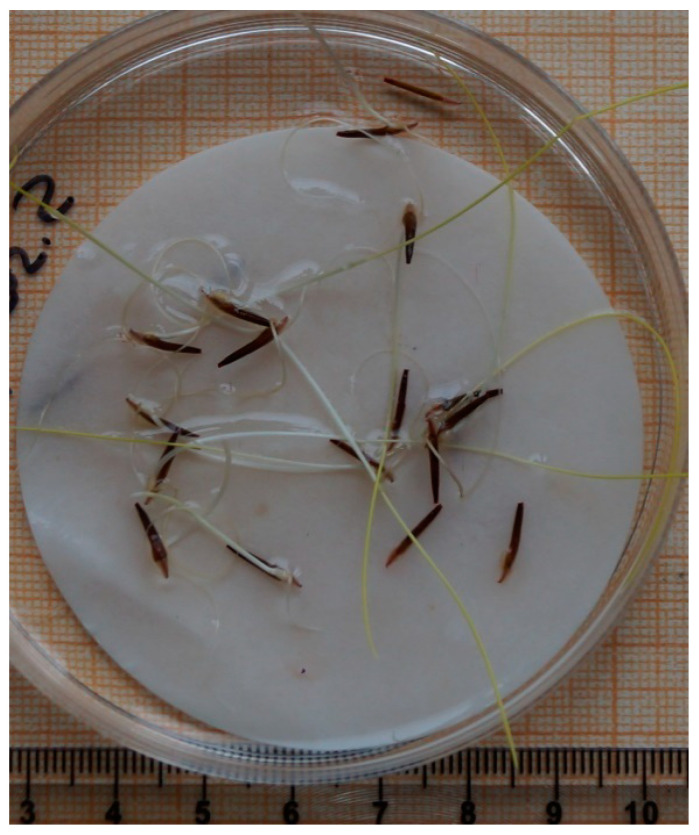
After 14 months of storage in liquid nitrogen, *S. sareptana* seeds were thawed and germinated within 30 days.

The third series of experiments evaluated the germination of *Stipa* L. seeds after 14 months of storage in liquid nitrogen. After thawing, *Stipa* seeds, except *S. sareptana*, did not germinate. However, all post-cryogenic seeds stained red-brown with formazan, indicating vigorous respiration. *S. ucrainica*, *S. tirsa*, *S. dasyphylla*, and *S. pulcherrima* germinated only after NaOH treatment. *S. adoxa* seeds did not germinate at all. *S. sareptana* seeds did not require NaOH treatment as they germinated within 30 days after thawing (Table 4, Figure 2). The third experiment indicated that the germination of all *Stipa* seeds, except *S. sareptana*, decreased during long-term cryopreservation. Cryopreservation as a stress factor could possibly contribute to increasing the depth of physiological dormancy of all studied *Stipa* species except *S. sareptana*. The third series of experiments also indicated that NaOH may be used as a germination stimulant for the *Stipa* seeds.

Most of the seeds from the control group stored in the dark at room temperature for 19 months lost germination and did not participate in further experiments.

#### 3.4.4. Experiment IV

The fourth series of experiments evaluated the germination of *Stipa* seeds after four years of cryopreservation. The germination of *Stipa* was only observed after 90 days and that of *S. adoxa* after 180 days. Germination of all *Stipa* seeds decreased significantly (Table 5). In this case, a longer germination period or a pre-treatment with stimulators is required to bring the seeds out of dormancy.

#### 3.4.5. Experiment V

The fifth series of experiments evaluated the effect of different stimulators on seed germination. As a result, we found that pre-treatment with KNO_3_ and GA_3_ did not cause the germination of seeds of all *Stipa* species except *S. sareptana*. These data suggest that treatment with KNO_3_ and GA_3_ led to the exit of the seeds from a non-deep physiological dormancy state. It may be assumed that the dormancy of seeds of all *Stipa* except *S. sareptana* was increased during cryopreservation. Pre-treatment of the seeds with H_2_O_2_ increased germination levels of *S. sareptana* and *S. dasyphylla* and stimulated germination of *S. ucrainica*. Pre-treatment with NaOH increased germination of *S. adoxa* and *S. pulcherrima* (Table 6). The effect of these stimulators was probably species dependent.

#### 3.4.6. Summary: Seed Germination after Cryopreservation

According to the summary data obtained for five years of seed cryopreservation, it can be concluded that short-term cryopreservation (14 days) stimulated their germination, but after two months of cryopreservation (70 days) germination of all *Stipa* seeds except *S. sareptana* decreased (Table 7). After four years of cryopreservation (1489 days), the dormancy of most *Stipa* except *S. sareptana* increased. Dormancy was cancelled either by stratification followed by prolonged cultivation (180–400 days) or by the use of the stimulators NaOH and H_2_O_2_ (Table 7). Perhaps a combination of cold stratification for more than 40 days and seed pre-treatment with NaOH or H_2_O_2_ would be an optimal stimulation of the seed germination.

## 4. Discussion

### 4.1. Cryopreservation of Stipa *L.*

Previously, three studies were publishedin which the authors investigated the effect of cryopreservation on *Stipa* seeds. Puchalski et al. (2014) showed that 92–100% of *S. pennata* seeds germinated after 30 days of storage in liquid nitrogen vapour (−160 °C) [55]. Levitskaya (2017b) stored *S. pennata* seeds at temperatures of 5 ± 1 °C (refrigerator), −20 ± 2 °C (freezer), and −196 °C (liquid nitrogen). After one month of storage, the germination rates of seeds stored at different temperatures did not decrease. After storage for 6 years, the germination of seeds stored at 5 ± 1 °C did not decrease, but the germination levels of seeds stored at −20 °C and −196 °C decreased several times [56]. Shevchenko et al. (2019) stored *S. capillata* seeds at room temperature, at −20 °C, and at −196 °C for 18 months. Then, the seed scales were cut with a scalpel, and the seeds were put on Petri dishes and germinated at 20 °C in the dark. The experiment showed that the seeds had low germination rates, both in the control (10.46 ± 7.45%) and after low temperature storage at −20 °C (12 ± 8.18%). The germination of *S. capillata* seeds increased significantly to 30.28 ± 10.73% after storage in liquid nitrogen [57].

Therefore, our data are in accordance with the published observations: short-term cryopreservation for 14 days stimulated initial germination (Table 2 and Table 7), as in Puchalski et al. (2014) and Levitskaya (2017b) [55,56]. Cryopreservation of seeds for about 1 year (415 days) and pre-treatment with 30% NaOH reliably stimulated germination of *S. sareptana* and *S. tirsa* (Table 4 and Table 7) seeds, as was reported in Shevchenko et al. (2019) [57]. Cryopreservation for more than 5 years significantly reduced germination of all *Stipa* species except *S. sareptana* and made it impossible without additional stimulation (Table 6 and Table 7), as was described in Levitskaya (2017b) [56].

### 4.2. Effect of Cryopreservation on Seed Germination

It is known that cryopreservation has different effects on the germination of seeds of even closely related plant species. For example, Nikishina et al. (2007) found that after 30 days of cryopreservation, seeds of *Dactylorhiza maculate* and *Platanthera bifolia* (L.) Rich. showed decreased germination, whereas for seeds of *D. fuchsii* (Druce) Soo, *D. incarnata*, and *D. baltica* (Klinge) Orlova stimulated germination was seen [58]. Liquid nitrogen treatment causes cracks to form in the seed-coat. As a result, the palisade layer of cells underlying the outer cuticle is disrupted and the seed-coat becomes more water permeable. Disruption of the seed-coat results in faster bulking and activation of intracellular metabolism [59]. It is also possible that cryopreservation, as a stress factor, may activate redox processes in the embryo and thus stimulate germination. In some cultivated species, a stimulating effect of cryopreservation has been observed, associated with the induction of enzyme complex activity [60]. In our experiments, after 70 days of cryopreservation, germination of all *Stipa* species except *S. sareptana* was reduced compared to the initial germination (Table 7). After 415 days of cryopreservation, germination was found only in seeds of *S. sareptana* that did not undergo additional stimulation (Table 4). At the same time, all the seeds were alive because they had been stained with TTX. After 1489 days of cryopreservation, all *Stipa* seeds germinated, butonly after 180 days (Table 5). Thus, the decrease in germination after cryopreservation progressed over time as a result of cumulative stress rather than initial stress. A similar phenomenon has been observed previously [41,61]. Walters et al., (2005) suggested that the mechanical structure of biopolymers is transformed when seeds are desiccated below critical moisture content. At cryogenic temperatures, intense intracellular molecular mobility is observed in over-desiccated seeds compared to more humid seeds. On this basis, it has been suggested that the combination of extreme desiccation and extreme cooling may result in anomalous temperature responses in aging kinetics [42]. This hypothesis is supported by Chmielarz’s (2009) research on the cryopreservation of *Prunus avium* L. seeds. The study found that seeds with more than 17% free water did not germinate, and those with less than 7% free water germinated less than 20%, while those with 9 to 16.9% free water showed maximum germination levels of more than 20% [62]. Levitskaya [43,61,63] conducted studies on the cryopreservation of seeds with different types of dormancy. The authors concluded that storage at freezing temperatures would be optimal for orthodox seeds of species growing in drought or frosty climates. These may be non-dormant seeds and seeds with organic dormancy of different types other than physiological. Seeds with deep physiological dormancy, adapted to storage in humid conditions, may be unstable to extreme drying and storage at freezing temperatures [43]. Using five *Campanula* species, Levitskaya (2015) revealed that the deeper the seed dormancy, the faster they aged at ultra-low temperatures. To explain this, the author suggested that seeds with and without physiological dormancy have different mechanisms involved in viability [63]. Perhaps, seeds with morphological and morphophysiological dormancy, as well as not-dormant seeds, survive in a dry state due to the general inhibition of metabolism as a consequence of the low hydration. They have evolved sHSPs (small heat shock proteins) and LEA-proteins (late embryogenesis abundant proteins), which are synthesized at late stages of seed maturation and are responsible for cell resistance to dehydration and dry storage, including these conditions at low temperatures. Seeds with physiological dormancy are able to maintain viability for a long time in a humid, hydrated state by repairing cytological damage through specific metabolism [64]. The cellular structure of such seeds is likely to change less during seed maturation and desiccation. It may be assumed that the development of seeds with physiological dormancy is terminated at an earlier stage, when low amounts of sHSPs and LEA, or less than the whole functional spectrum of these proteins, are synthesized [43]. Therefore, before cryopreservation, it is necessary to find out whether seeds are in dormancy, to determine the type of dormancy, to select the methods of dormancy removal, and the conditions of seed germination.

### 4.3. Effect of Dry Storage and Cold Stratification on Stipa Seed Germination

At the beginning of our experiment, the seeds were stored in the dark at room temperature for three months after their collection. Only *S. sareptana* germinated after 14 days and had a DI of 0.6. The other *Stipa* seeds germinated after one month and had a DI of 1. Therefore, they had a deeper physiological dormancy than *S. sareptana* from the beginning of the experiment. All *Stipa* seeds were in the state of organic dormancy [53]. According to Nikolaeva (1985), other species of *Stipa*: S. *bigeniculata* D. K. Hughes, *S. nitida* V. *S. Summerhayes* et S. E. Hubbard, and *S. viridula* Trin., do not have a deep physiological dormancy [65]. According to Hu et al. (2014), *S. bungeana* also has a non-deep physiological dormancy [66]. Since we were not able to find any information, we assumed the same non-deep physiological dormancy for the *Stipa* we studied. This type of dormancy is due to the low growth activity of the embryo which, combined with the poor gas permeability of the seed-coat, provides a dual or physiological mechanism for inhibiting germination [67,68]. To test this, we cut seed scales. This stimulated the germination of all living embryos, confirming our suggestion about non-deep physiological dormancy of *Stipa* seeds studied.

In addition to damaging the scales, dry storage for several months, short cold stratification, or treatment of the seeds with phytohormones gibberellins (GA_3_, GA_4+7_) and cytokinins (kinetin, zeatin, benzylaminopurin) are recommended to break out of the non-deep physiological dormancy [69]. According to the data available in the literature, germination of *Stipa* under laboratory conditions may vary considerably. For example, Ronnenberg et al. (2008) reported that the field germination levels of *S. krylovii* was 3%, *S. glareosa*—0.6%, and *S. gobica*—0.1%. In addition, the three-year-old seeds were germinatingin the field on the third year after very heavy rain. The laboratory germination of freshly collected seeds of all three species studied at 20/10 °C was 90%. Dry storage of seeds for six months did not change germination levels. Cold stratification increased the germination only of *S. krylovii* [70]. Hu et al. (2014) reported that the highest germination rates under laboratory conditions were 25% for fresh *S. bungeana* seeds in the dark at 20 °C. After storage of the seeds in the dark for six months at 5 °C, germination increased from 25% to 63%, and after storage of the seeds at 20 °C, germination increased from 25% to 69% [66]. Zhang et al. (2017) observed that the laboratory germination of fresh *S. bungeana* seeds in 2015 ranged from 13.7% to 41.2% at 10/20 °C, 17.4% to 73.4% at 15/25 °C, and 0% to 0.7% at 20/30 °C. After six months of dry storage, the average germination of *S. bungeana* seeds from the eight populations increased. However, germination varied between populations. In some populations, germination rates decreased [71]. Krichen et al. (2017) reported that the germination under laboratory conditions of *S. tenacissima* seeds at 20 °C was 50%. After cold stratification, the germination of *S. tenacissima* seeds increased from 50% to 70% depending on the length of stratification [72]. Nozdrina et al. (2021) found that germination of *S. capillata* seeds in the laboratory was 16%, and of *S. pennata* L.—4%. After stratification, the germination of *S. capillata* L. increased almost 3-fold and reached 45%, whereas the germination of *S. pennata* L. did not change [73]. In the above examples, the effect of cold stratification on germination of *Stipa* seeds is ambiguous, and probably depends on the conditions and duration of stratification and the nature of species used.The relatively low germination rate of wild species may be attributed to the seeds’ ability to germinate at varying times due to the presence of distinct types of organic dormancy. As a result, a seed bank is formed in the soils, which serves as a backup in times of difficult growing years [69]. Furthermore, germination depends on weather conditions in the year of reproduction, population growth conditions, i.e., seed formation conditions, seed germination conditions, and seed genetics [56,71,74].

In our second experiment without pre-treatment (stratification and stimulators), after eight months of storage at room temperature in dark, seed germination rates varied from 0% to 55% (average 14%) (Table 3). The best germination was observed in *S. sareptana*, while seeds of *S. adoxa* and *S. pulcherrima* did not germinate (Table 3). Most of the seeds of *S. sareptana* were probably in a non-deep physiological dormancy, while the seeds of the other *Stipa* investigated had a deeper physiological dormancy. In the first experiment, after six months of storage at room temperature in the dark and 42 days of stratification, seed germination rates ranged from 21% to 57% and averaged 40% (Table 2). In the second experiment, after eight months of storage at room temperature in the dark and 21 days of stratification, seed germination varied from 0% to 79%, with an average of 23% (Table 3). After stratification, the germination of *S. sareptana*, *S. dasyphylla*, and *S. pulcherrima* increased. However, the germination of *S. ucrainica* and *S. tirsa* did not change, and *S. adoxa* did not germinate (Table 3). The decrease in average germination in the second experiment compared to the first was most likely due to the reduction in stratification by half. The cold stratification usually promotes seed emergence from physiological dormancy, and the deeper the dormancy, the longer the stratification period should be [68,69].

On the one hand, stratification can soften the seed-coat and improve its permeability. On the other hand, the process of stratification changes the physiological parameters of the seed: enzyme activity increases, hormonal balance changes, nucleic acids are synthesized, and all metabolic processes are activated. At low temperatures, the initial changes necessary for the synthesis of gibberellins also take place [69]. As cold stratification stimulates the synthesis of endogenous gibberellins, treatment of seeds with exogenous gibberellins may replace cold stratification, as well as dry storage and light, to emerge from non-deep physiological dormancy [75].

### 4.4. Effect of GA_3_ on Stipa Germination

According to Naylor and Simpson (1961), gibberellins have a dual effect. Firstly, at low concentrations (0.5 mg/L), they stimulate embryo metabolism. Secondly, at much higher concentrations (50 mg/L), they stimulate endosperm enzymes and promote the activity of hydrolytic enzymes in the aleurone layer of barley seeds [76]. The main function in starch hydrolysis in the endosperm is performed by α- and β-amylases. The α-amylases hydrolyze starch into oligosaccharides. β-amylases convert oligosaccharides into maltose [77]. α-amylase gene expression is regulated by the gibberellin-dependent transcription factor GAMyb [78]. As a result, gibberellins secreted by the embryo initiate the expression of α-amylase genes in the aleurone layer. This leads to the lysis of starch granules in the endosperm and provides the young seedling with food substances [79]. Thus, GAs are widely used to break the non-deep physiological dormancy of seeds [80]. Hu et al. (2014) found that treatment of fresh *S. bungeana* seeds with GA_3_ increased germination by 11% (from 25% to 36%) [66]. In our experiments, germination of *S. sareptana* seeds increased by 25% (from 35% to 70%) after treatment with GA_3_ (Table 6), while other *Stipa* seeds did not germinate at all. According to Nikolaeva et al. (1999), GA_3_, as a rule, has no effect on intact seeds with deep dormancy, even after partial stratification [69]. This confirms our assumption that *S. sareptana* has a non-deep physiological dormancy, while other *Stipa* species have deeper physiological dormancy.

### 4.5. Effect of KNO_3_ on Stipa Germination

Nitrate is a prevalent inorganic ion in soils. It has the ability to decrease seed dormancy and promote seed germination [65,80]. Previously, potassium nitrate has been widely used to break the dormancy of cereal seeds [81]. Nitrate promotes germination by binding NIN-like protein 8 to the CYP707A2 promoter and activating its expression, thus reducing ABA levels after germination [82]. Nitrate treatment (KNO_3_) enhances seed quality potentially, and most likely, due to the fact that K^+^ at optimum concentration in KNO_3_ is used as a catalyst to enhance the metabolic activities of adenosine triphosphatase (ATPase) and nicotinamide dinucleotide (NAD). Additionally, K^+^ promotes biosynthesis and the regulation of auxin activity in seeds [83]. According to Roberts and Smith, (1977) the non-deep physiological dormancy of seeds may be disturbed by nitrite and nitrate (more often KNO_3_) as hydrogen acceptors [84]. Hu et al. (2014) found that treatment of fresh *S. bungeana* seeds with 1 mM KNO_3_ for 14 days increased germination by 19% (from 25% to 44%) [66]. In our own experiments, germination of *S. sareptana* seeds increased by 18% (from 35% to 63%) after KNO_3_ treatment, while other *Stipa* seeds did not germinate under these conditions (Table 6). This also confirms our assumption that *S. sareptana* has a non-deep physiological dormancy, while other *Stipa* species have deeper physiological dormancy.

### 4.6. Effect of NaOH on Stipa Germination

Seed treatment with strong acids or alkalis promotes scarification, i.e., damage to the seed-coat for access to oxygen and water [65]. If seed dormancy is associated with synthesis of inhibitors by the seed-coat, NaOH may promote the washing of these inhibitors from the embryo covering tissues [66]. Hu et al. (2014) found that the treatment of fresh *S. bungeana* seeds with 30% NaOH for 20, 40, and 60 min increased germination rates from 25% to 45%; and 63% and 82%, respectively [66]. In our own experiments, only NaOH treatment stimulated seed germination of *S. ucrainica*, *S. tirsa*, *S. dasyphylla*, and *S. pulcherrima* (Table 4). In the following experiments, germination of all *Stipa* seeds, except *S. ucrainica*, was observed after NaOH treatment (Table 6).

### 4.7. Effect of H_2_O_2_ on Stipa Germination

Peroxide, as well as other inorganic reagents, is able to effectively modify the permeability of the seed-coat by scarification and thus break dormancy. Furthermore, H_2_O_2_ acts as a respiratory inhibitor, while simultaneously promoting the pentose phosphate pathway, which plays an important role in seed germination [84]. The activation of certain respiratory inhibitors may promote the germination of dormant seeds by suppressing cytochrome oxidase or catalase activity, leading to increased NADPH oxidase activity and, consequently, the induction of the pentose phosphate pathway. These resultson the reoxidation of NADPHxH, as suggested by Roberts and Smith, could be important for dormancy breaking. However, the function of NADPH in seed germination remains unclear. It is believed that NADPH plays a significant role in reductive reactions [84]. In the current study, all *Stipa* seeds, except for *S. pulcherrima*, germinated after treatment with H_2_O_2_ (Table 6).

It is possible that pre-treatment stimulators act in a species-dependent manner. In the case of the *Stipa* seeds studied, prolonged stratification (more than 40 days) combined with H_2_O_2_ or NaOH pretreatment would be the optimal to stimulate seed germination (Table 7).

### 4.8. The Ability to Cryopreservation Is Species-Dependent

It is interesting to note that *S. sareptana*, the most widespread of the *Stipa* species studied, showed high germination rates and the best cryopreservation ability. Habitats of *S. sareptana* have been recorded in a widespread area from the south of the Nizhny Novgorod region from Russia to Tajikistan and from the eastern border of Kazakhstan to the west of the Rostov region of Russia. According to Lavrenko (1993), *S. sareptana* is the dominant formation in the deserted steppes of the Zavolzhsko–Kazakstan province [85]. *S. sareptana* communities occupy large areas in almost all habitat types and are landscape-scale in this province, especially east of the Ural River [85,86]. According to Tsvelev (2012), *S. sareptana* belongs to the *Leiostipa* section, is related to *S. capillata* [87], and is a xerophilous dense turfgrass [86]. The other *Stipa* species studied in this work belong to the Eurasian section *Stipa*, are related to *S. pennata* [87], and are mesoxerophilic turfgrasses native to meadows and true steppes [86]. It is probably that the characteristics that differentiate *S. sareptana* from other *Stipa* species, such as its greatest drought tolerance, lowest seed weight with the least amount of free water in it (unpublished data), and non-deep physiological dormancy, contribute to its survival under more extreme and stressful conditions and, therefore, correlate with its greatest resistance to cryostress.

## 5. Conclusions

As a result of the conducted research, the authors came to the following conclusions: all *Stipa* seeds studied were viable and had different germination rates depending on the species. *S. sareptana* had a non-deep physiological dormancy, while the other *Stipa* species studied had a deeper dormancy at the start. As germination of about half of the *Stipa* species seeds was stimulated by short-term cryopreservation and removal of the seed-coats, the seeds were in a non-deep physiological dormancy. Since germination of *S. sareptana* seeds was stimulated by KNO_3_ and GA_3_, its seeds were in a non-deep physiological dormancy. As germination of other *Stipa* species was not stimulated by KNO_3_ and GA_3_, their germination decreased over time; perhaps cryopreservation increased the depth of their physiological dormancy. It is possible that cryopreservation may have a negative effect on the germination of some orthodox seeds. NaOH treatment contributed to germination of *S. adoxa* and *S. pulcherrima* seeds after 5 years of cryopreservation. H_2_O_2_ treatment contributed to germination of *S. sareptana*, *S. ucrainica* and *S. dasyphylla* seeds after 5 years of cryopreservation.

We believe it is necessary to continue research focused on the conservation of *Stipa* seeds by studying the seeds collected at different years, and also test the seeds with various moisture contents and stored at different temperatures. Further research is needed to minimize germination loss during seed storage. It would also be interesting to find out why some orthodox seeds are sensitive to cryopreservation and how this can be overcome.

## Figures and Tables

**Table 1:** Coordinates of the collection site of the *Stipa* samples.

№	Species	Latitude	Longitude
1	*S. sareptana*	47°23′57.3″	39°65′23.9″
2	*S. ucrainica*	47°23′46.1″	39°65′61.0″
3	*S. tirsa*	47°23′78.2″	39°65′70.1″
4	*S. dasyphylla*	47°23′54.5″	39°65′71.5″
5	*S. adoxa*	47°23′47.7″	39°65′83.3″
6	*S. pulcherrima*	47°23′69.3″	39°66′00.6″

The number in the table corresponds to the number on the map. This number is the location of the Stipa seeds collection site.

**Table 2:** Effect of short-term cryopreservation on germination and DI levels of *Stipa* seeds.

Species	Control Group Room t + Stratification 42 Days		Cryo Group N_2_ (−196 °C) 14 Days + Stratification 42 Days	
	Seed Germination, %	DI	Seed Germination, %	DI
*S. sareptana*	57	0.43	84	0.16
*S. ucrainica*	43	0.57	59	0.41
*S. tirsa*	32	0.68	50	0.50
*S. dasyphylla*	28	0.72	44	0.56
*S. adoxa*	21	0.79	50	0.50
*S. pulcherrima*	56	0.44	69	0.31
average, %	40	0.60	59	0.41

**Table 3:** Effect of stratification and cryopreservation on germination of *Stipa* seeds.

Species	Seed Germination, %			
	Control Group		Cryo Group	
	RT (8th Months) No Stratification	RT (7th Months) + Stratification 21 Days	N_2_ (−196 °C) 91 Days No Stratification	N_2_ (−196 °C) 70 Days + Stratification 21 Days
*S. sareptana*	55	79	83	100
*S. ucrainica*	8	8	12	13
*S. tirsa*	10	10	11	17
*S. dasyphylla*	13	22	19	27
*S. adoxa*	0	0	10	10
*S. pulcherrima*	0	20	9	50
average, %	14	23	24	36

**Table 4:** Effect of pre-treatment with NaOH on germination of *Stipa* seeds.

Species	Seed Germination, %	
	No Pre-Treatment	NaOH
*S. sareptana*	90	-
*S. ucrainica*	0	17
*S. tirsa*	0	57
*S. dasyphylla*	0	29
*S. adoxa*	0	0
*S. pulcherrima*	0	29
average, %	15	26

**Table 5:** Effect of germination duration of *Stipa* seeds.

Species	Seed Germination, %			
	30 Days	90 Days	180 Days	400 Days
*S. sareptana*	35	59	61	61
*S. ucrainica*	0	5	16	26
*S. tirsa*	0	12	24	35
*S. dasyphylla*	0	7	7	14
*S. adoxa*	0	0	8	25
*S. pulcherrima*	0	20	30	30
average, %	6	17	24	32

**Table 6:** Effect of GA_3_, KNO_3_, NaOH, and H_2_O_2_ stimulators on germination levels of *Stipa* seeds.

Species		Seed Germination, %			
	No Stimulator	GA_3_	KNO_3_	NaOH	H_2_O_2_
*S. sareptana*	35	70	63	56	90
*S. ucrainica*	0	0	0	0	7
*S. tirsa*	-	-	-	-	-
*S. dasyphylla*	0	0	0	6	11
*S. adoxa*	0	0	0	13	6
*S. pulcherrima*	0	0	0	7	0
average, %	7	14	13	16	23

**Table 7:** Summary: factors (duration of cryopreservation, presence and duration of stratification, treatment with stimulators, and duration of germination) affecting the germination of *Stipa* seeds.

	Conditions and Duration of the Experiment						
Cryopreservation N_2_	Control	14 Days	70 Days	415 Days	1489 Days	1951 Days	1951 Days
**stimulator**	**-**	**-**	**-**	**NaOH**	**-**	**NaOH**	**H_2_O_2_**
**stratification**	**42 days**	**42 days**	**21 days**	**-**	**30 days**	**-**	**-**
**germination**	**30 days**	**30 days**	**30 days**	**30 days**	**400 days**	**30 days**	**30 days**
**Species**	**Seed germination, %**						
*S. sareptana*	57	84	100	90	61	56	90
*S. ucrainica*	43	59	13	17	26	0	7
*S. tirsa*	32	50	17	57	35	-	-
*S. dasyphylla*	28	44	27	29	14	6	11
*S. adoxa*	21	50	10	0	25	13	6
*S. pulcherrima*	56	69	50	29	30	7	0
average, %	40	59	36	37	32	16	23

## Data Availability

The data presented in this study are available upon request from the corresponding author.

## References

[1] Sakai A. (1965). Survival of plant tissue at super-low temperature III. Relation between effective prefreezing temperatures and the degree of frost hardiness. Plant Physiol..

[2] Reed B.M. (2017). Plant cryopreservation: A continuing requirement for food and ecosystem security. Vitr. Cell. Dev. Biol..

[3] Panis B. (2019). Sixty Years of Plant Cryopreservation: From Freezing Hardy Mulberry Twigs to Establishing Reference Crop Collections for Future Generations. Acta Hortic..

[4] Streczynski R., Clark H., Whelehan L.M., Ang S.-T., Hardstaff L.K., Funnekotter B., Bunn E., Offord C.A., Sommerville K.D., Mancera R.L. (2019). Current issues in plant cryopreservation and importance for ex situ conservation of threatened Australian native species. Aust. J. Bot..

[5] Botanic Gardens Conservational International. https://www.bgci.org/our-work/saving-plants/seed-conservation/.

[6] Kaczmarczyk A., Funnekotter B., Menon A., Phang P.Y., Al-Hanbali A., Bunn E., Mancera R.L., Katkov I.I. (2012). Current issues in plant cryopreservation. Current Frontiers in Cryobiology.

[7] Sakai A., Kobayashi S., Oiyama I. (1991). Survival by vitrification of nucellar cells of navel orange (*Citrus sinensis* var. brasiliensis Tanaka) cooled to −196 °C. J. Plant Physiol..

[8] Matsumoto T., Mochida K., Itamura H., Sakai A. (2001). Cryopreservation of persimmon (*Diospyros kaki* Thunb.) by vitrification of dormant shoot tips. Plant Cell Rep..

[9] Steponkus P.L., Zaitlin M., Day P., Hollander A. (1985). Fundamental aspects of cryoinjury as related to cryopreservation of plant cells and organs. Biotechnology in Plant Science.

[10] Kim H.-H., Lee Y.-G., Shin D.-J., Ko H.-C., Gwag J.-G., Cho E.-G., Engelmann F. (2009). Development of alternative plant vitrification solutions in droplet-vitrification procedures. Cryo Lett..

[11] Fabre J., Dereuddre J. (1990). Encapsulation-dehydration: A new approach to cryopreservation of Solanum shoot-tips. Cryo Lett..

[12] Hirai D., Shirai K., Shirai S., Sakai A. (1998). Cryopreservation of in vitro grown meristems of strawberry (*Fragaria* × *ananassa* Duch.) by encapsulation-vitrification. Euphytica.

[13] Hirai D., Sakai A. (1999). Cryopreservation of in vitro-grown meristems of potato (*Solanum tuberosum* L.) by encapsulation-vitrification. Potato Res..

[14] Volk G.M., Maness N., Rotindo K. (2004). Cryopreservation of garlic (*Allium sativum* L.) using plant vitrification solution 2. Cryo Lett..

[15] Panis B., Piette B., Swennen R. (2005). Droplet vitrification of apical meristems: A cryopreservation protocol applicable to all *Musaceae*. Plant Sci..

[16] Yamamoto S., Rafique T., Priyantha W.S., Fukui K., Matsumoto T., Niino T. (2011). Development of a cryopreservation procedure using aluminium cryo-plates. Cryo Lett..

[17] Yamamoto S., Rafique T., Fukui K., Sekizawa K., Niino T. (2012). V-cryo-plate procedure as an effective protocol for cryobanks: Case study of mint cryopreservation. Cryo Lett..

[18] Nadarajan J., Pritchard H.W. (2014). Biophysical characteristics of successful oilseed embryo cryoprotection and cryopreservation using vacuum infiltration vitrification: An innovation in plant cell preservation. PLoS ONE.

[19] Funnekotter B., Whiteley S.E., Turner S.R., Bunn E., Mancera R.L. (2015). Evaluation of the new vacuum infiltration vitrification (VIV) cryopreservation technique for native Australian plant shoot tips. Cryo Lett..

[20] Funnekotter B., Bunn E., Mancera R.L. (2017). Cryo-mesh: A simple alternative cryopreservation protocol. Cryo Lett..

[21] Engelmann F. (2004). Plant cryopreservation: Progress and prospects. Vitr. Cell. Dev. Biol.-Plant.

[22] Burke M.J., Gusta L.V., Quamme H.A., Weiser C.J., Li P.H. (1976). Freezing and injury in plants. Annu. Rev. Plant Physiol..

[23] Steponkus P.L. (1984). Role of the plasma membrane in freezing injury and cold acclimation. Annu. Rev. Plant Physiol..

[24] Gordon-Kamm W.J., Steponkus P.L. (1984). Lamellar-to-hexagonal II phase transitions in the plasma membrane of isolated protoplasts after freeze-induced dehydration. Proc. Natl. Acad. Sci. USA.

[25] Helliot B., Swennen R., Poumay Y., Frison E., Lepoivre P., Panis B. (2003). Ultrastructural changes associated with cryopreservation of banana (*Musa* spp.) highly proliferating meristems. Plant Cell Rep..

[26] Wesley-Smith J., Walters C., Pammenter N.W., Berjak P. (2015). Why is intracellular ice lethal? A microscopical study showing evidence of programmed cell death in cryo-exposed embryonic axes of recalcitrant seeds of *Acer saccharinum*. Ann. Bot..

[27] Levitt J. (1962). A sulfhydryl-disulfide hypothesis of frost injury and resistance in plants. J. Theor. Biol..

[28] Benson E.E. (1990). Free Radical Damage in Stored Plant Germplasm.

[29] Barry H., Gutteridge J.M.C. (2015). Free Radicals in Biology and Medicine.

[30] Wise R.R. (1995). Chilling-enhanced photooxidation: The production, action and study of reactive oxygen species produced during chilling in the light. Photosynth. Res..

[31] Skyba M., Urbanova M., Kapchina-Toteva V., Kosuth J., Harding K., Cellarova E. (2010). Physiological, biochemical and molecular characteristics of cryopreserved *Hypericum perforatum* L. shoot tips. Cryo Lett..

[32] Benson E.E., Bremner D., Fuller B.J., Lane N., Benson E.E. (2004). Oxidative stress in the frozen plant: A free radical point of view. Life in the Frozen State.

[33] Reed B.M. (2014). Antioxidants and cryopreservation, the new normal?. Acta Hortic..

[34] Ren L., Wang M.-R., Wang Q.-C. (2021). ROS-induced oxidative stress in plant cryopreservation: Occurrence and alleviation. Planta.

[35] Harding K., Johnston J.W., Benson E.E. (2009). Exploring the physiological basis of cryopreservation success and failure in clonally propagated in vitro crop plant germplasm. Agric. Food Sci..

[36] Jiang X., Ren R., Di W., Jia M., Li Z., Liu Y., Gao R. (2019). Hydrogen peroxide and nitric oxide are involved in programmed cell death induced by cryopreservation in *Dendrobium* protocorm-like bodies. Plant Cell Tissue Organ. Cult..

[37] Thomashow M.F. (1999). Plant cold acclimation: Freezing tolerance genes and regulatory mechanisms. Annu. Rev. Plant Physiol. Plant Mol. Biol..

[38] Pritchard H.W., Nadarajan J., Reed B.M. (2008). Cryopreservation of orthodox (desiccation tolerant) seeds. Plant Cryopreservation: A Practical Guide.

[39] Silva R.L., Souza E.H., Vieira L.J., Pelacany C.R., Souza F.V.D. (2017). Cryopreservation of pollen of wild pineapple accessions. Sci. Hortic..

[40] Stanwood P.C., Bass L.N., Li P., Sakai A. (1978). Ultracold preservation of seed germplasm. Plant Cold Hardiness and Freezing Stress.

[41] Walters C., Hill L.M., Wheeler L.J. (2005). Dying while dry: Kinetics and mechanisms of deterioration in desiccated organisms. Integr. Comp. Biol..

[42] Ballesteros D., Walters C. (2011). Detailed characterization of mechanical properties and molecular mobility within dry seed glasses: Relevance to the physiology of dry biological systems. Plant J..

[43] Levitskaya G.E. (2017). The influence of the storage temperature on the seeds of wild species. 3. Seeds with morphological and morphophysiological dormancy. Plant Resour..

[44] Joubert D.C., Small J.G.C. (1982). Seed germination and dormancy of *Stipa trichotoma* (*Nassella* Tussock). Part 1. Effect of dehulling, constant temperatures, light, oxygen, activated charcoal and storage. S. Afr. J. Bot..

[45] Gasque M., García-Fayos P. (2003). Seed dormancy and longevity in *Stipa tenacissima* L. (Poaceae). Plant Ecol..

[46] Mordkovich V.G. (2014). Stepnye Jekosistemy.

[47] Xiaomin L.V., Guangsheng Z., Yuxui W., Song X. (2016). Sensitive indicators of *Stipa* species to changiang temperature and precipitation in inner Mongolia grassland, China. Front. Plant Sci..

[48] Xiaomin L.V., Guangsheng Z. (2018). Climatic suitability of the geographic distribution of *Stipa breviflora* in Chinese temperate grassland under climate change. Sustainability.

[49] Tishkov A.A., Belonovskaya E.A., Zolotukhin N.A., Titova S.V., Tsarevskaya N.G., Chendev Y.G. (2020). Preserved Sections of Steppes as the Basis for the Future Ecological Framework of Belgorod Oblast. Arid. Ecosyst..

[50] Sprinchanou E.K., Antipin M.I., Vysotskaya O.N. The germination of six needlegrass species (*Stipa* L.) before and after cryopreservation. Proceedings of the XIth International Conference “The Biology of Plant Cells In Vitro and Biotechnology”.

[51] Potapova N.A., Nazyrova R.I., Elmanov S.A., Moshnyaga O.V., Ganitsky I.V., Mirutenko M.V., Vilyaeva N.A., Ripa S.I., Milyutina M.L., Fedotov M.P. (2019). Regional and Local Protected Areas of the Russian Federation.

[52] Botanic Garden of the Southern Federal University (Rostov-na-Donu). https://bg.sfedu.ru.

[53] Rubida A.L. (1987). Determining Green Needlegrass (*Stipa viridula* Trin.) Seed Germination and Viability. Electronic Theses and Dissertations. https://openprairie.sdstate.edu/etd/4472.

[54] Offord C.A., Mckensy M.L., Cuneo P.V. (2004). Critical review of threatened species collections in the NSW Seedbank: Implications for ex situ conservation of biodiversity. Pac. Conserv. Biol..

[55] Puchalski J., Kapler A., Niemczyk M., Walerowski P., Krzyżewski A., Nowak A., Podyma W. (2014). Long-term seed cryopreservation of rare and endangered Polish Ponto-Panonian plant species. Opole Sci. Soc. Nat. J..

[56] Levitskaya G.E. (2017). Rare species in experimental collection of cryobank wilding seeds in the Institute of Cell Biophysics of RAS. Tambov. Univ. Rep. Ser. Nat. Tech. Sci..

[57] Shevchenko N., Shyriaieva D., Kovalenko G. (2021). Germination of *Stipa Capillata* L. before and after low temperature storage. Cryo Lett..

[58] Nikishina T.V., Popova E.V., Popovich E.A., Shumilov V.Y., Popov A.S., Vakhrameeva M.G., Varlygina T.I., Kolomeitseva G.L., Burov A.V., Shirokov A.I. (2007). Cryopreservation of seeds and protocorms of rare temperate orchids. Russ. J. Plant Phys..

[59] Levitskaya G.E. (2009). The biological characteristics of seeds of some species of the flora of the southern of Moscow region and their response to cryoconservation. Plant Resour..

[60] Stribul T.F. (1993). Effect of Low Temperatures on Initial Growth Intensity and Productive Properties of Maize and Vegetable Seeds. Ph.D. Thesis.

[61] Levitskaya G.E. (2014). The influence of the storage temperature on the seeds of wild species. 1. The not-dormant seeds and seeds with non-deep physiological dormancy. Plant Resour..

[62] Chmielarz P. (2009). Cryopreservation of dormant orthodox seeds of forest trees: Mazzard cherry (*Prunus avium* L.). Ann. For. Sci..

[63] Levitskaya G.E. (2015). The influence of the storage temperature on the seeds of wild species. 2. seeds with physiological dormancy in the case of *Campanula* (Campanulaceae) species. Plant Resour..

[64] Villiers T.A. (1972). Cytological study in dormancy. II. Pathological ageing changes during prolonged dormancy and recovery upon dormancy release. New Phytol..

[65] Nikolaeva M.G., Razumova M.V., Gladkova V.N., Danilova M.F. (1985). Reference Book on Dormant Seed Germination.

[66] Hu X.W., Wu Y.P., Ding X.Y., Zhang R., Wang Y.R., Baskin J.M., Baskin C.C. (2014). Seed dormancy, seedling establishment and dynamics of the soil seed bank of *Stipa bungeana* (Poaceae) on the loess plateau of northwestern China. PLoS ONE.

[67] Nikolaeva M.G., Khan A.A. (1982). Factors affecting the seed dormancy pattern. The Physiology and Biochemistry of Seed Development, Dormancyand Germination.

[68] Baskin C., Baskin J.M. (2014). Seed Ecology, Biogeography, and Evolution of Dormancy and Germination.

[69] Nikolaeva M.G., Ljanguzova I.V., Pozdova L.M. (1999). Biologija Semjan.

[70] Ronnenberg K., Wesche K., Hensen I. (2008). Germination ecology of Central Asian *Stipa* spp.: Differences amongspecies, seed provenances, and the importance of field studies. Plant Ecol..

[71] Zhang R., Baskin J.M., Baskin C.C., Mo Q., Chen L., Hu X., Wang Y. (2017). Effect of population, collection year, after-ripening andincubation condition on seed germination of *Stipa bungeana*. Sci. Rep..

[72] Krichen K., Vilagrosa A., Chaieb M. (2017). Environmental factors that limit *Stipa tenacissima* L. germination and establishment in mediterranean arid ecosystems in a climate variability context. Acta Physiol. Plant.

[73] Nozdrina M.A., Voldaeva S.Y., Volkova E.M., Rozova I.V. (2021). The studying of seeding quality of steppe plant species. Izv. TulSU. Nat. Sci..

[74] Hodgkin T., Hegarty T.W. (1978). Genetically determined variation in seed germination and field emergence of *Brassica oleracea*. Ann. Appl. Biol..

[75] Razumova M.V., Nikolaeva M.G. (1981). Rol’ Temperatury i Fitogormonov v Narushenii Pokoja Semjan.

[76] Naylor J.M., Simpson G.M. (1961). Dormancy studies in seed of avena fatua: 2. a gibberellin-sensitive inhibitory mechanism in the embryo. Canad. J. Bot..

[77] Bilderback D.E. (1973). A Simple Method to Differentiate between α- and β-Amylase. Plant Physiol..

[78] Gubler F., Kalla R., Roberts J.K., Jacobsen J.V. (1995). Gibberellin-regulated expression of amybgene in barleyaleurone cells: Evidence for Myb transactivation of a high-pIα-amylase gene promoter. Plant Cell.

[79] Gubler F., Watts R.J., Kalla R., Matthews P., Keys M., Jacobsen J.V. (1997). Cloning of a rice cDNA encoding a transcription factor homologous to barley GAMyb. Plant Cell Physiol..

[80] Baskin C.C., Baskin J.M. (2014). Seeds: Ecology, Biogeography and Evolution of Dormancy and Germination.

[81] International Seed Testing Association (2012). International Rules for Seed Testing. Seed Science and Technology 27 (Supplement). https://www.seedtest.org/en/.

[82] Sano N., Marion-Poll A. (2021). ABA Metabolism and homeostasis in seed dormancy and germination. Int. J. Mol. Sci..

[83] Gattward J.N., Almeida A.A.F., Souza J.J.O., Gomes F.P., Kronzucker H.J. (2012). Sodium–potassium synergism in *Theobroma Cacao*: Stimulation of photosynthesis, water-use efficiency and mineral nutrition. Physiol. Plant.

[84] Roberts E.H., Smith R.D., Khan A.A. (1977). Pentose phosphate pathway and germination. The Physiology and Biochemistry of Seed Dormancy and Germination.

[85] Lavrenko E.M., Karamysheva Z.V., Coupland R.T. (1993). Steppes of the former Soviet Union and Mongolia. Ecosystems of the World.

[86] Lavrenko E.M. (1991). Stepi Evrazii.

[87] Tzvelev N. (2012). Notes on the tribe *Stipeae* Dumort. (*Poaceae*). Novit. Syst. Plant. Vasc..

